# The application of radiomics in vestibular schwannomas

**DOI:** 10.1017/S0022215125000258

**Published:** 2025-08

**Authors:** Terrenjit Gill, David Hamilton, Amarkumar Rajgor

**Affiliations:** 1 GKT School of Medical Education, Faculty of Life Sciences and Medicine, King’s College London, London, UK; 2 Newcastle University, Newcastle upon Tyne, UK; 3 Freeman Hospital, Newcastle upon Tyne Hospitals NHS Foundation Trust, Newcastle upon Tyne, UK; 4 Population Health Sciences Institute, National Institute for Health & Care Research, Newcastle University, Newcastle upon Tyne, UK; 5 Department of Otolaryngology – Head and Neck Surgery, Newcastle upon Tyne Hospitals NHS Foundation Trust, Newcastle upon Tyne, UK

**Keywords:** radiomics, artificial intelligence, neuroma, acoustic, treatment outcome, head and neck neoplasms

## Abstract

**Objectives:**

Radiomics refers to converting medical images into high-quality quantitative data. This review examines applications of radiomics in vestibular schwannomas and future considerations for translation into clinical practice.

**Methods:**

The review was pre-registered on Prospero (ID: CRD42024579319). A comprehensive systematic review-informed search of the Ovid Medline, Embase and Global Health online databases was undertaken using the keywords ‘acoustic neuroma’ or ‘vestibular schwannoma’ or ‘cerebellopontine angle tumour’ or ‘cerebellopontine tumour’ or ‘head and neck cancer’ were combined with ‘radiomic’ or ‘signature’ or ‘machine learning’ or ‘artificial intelligence’.

**Results:**

The studies (*n* = 6) were categorised into two groups: radiomics for pre-operative decision-making (*n* = 1) and radiomics for treatment outcomes (*n* = 5). Radiomic features were significantly associated with clinical outcomes. Radiomics-based predictive models were superior to expert vision.

**Conclusion:**

Radiomics has potential for improving multiple aspects of vestibular schwannoma care, but lack of studies inhibited firm conclusions. Prospective studies are required to progress this field.

## Introduction

Vestibular schwannomas, also known as acoustic neuromas, are benign tumours that form along the vestibulocochlear cranial nerve.^1^ Whilst rare, they account for 8 per cent of all primary brain tumours.^2^ Given the role of this nerve in both hearing and balance, symptoms can be truly debilitating for patients and can have a detrimental impact on their quality of life. Thus, newer and more effective methods of detection and decision-making for vestibular schwannomas are necessary.

**Figure 1.**
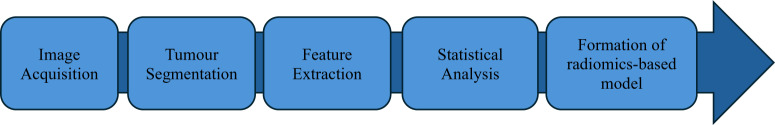
Radiomics Workflow Adopted from Rajgor et al.

In the diagnostic process of vestibular schwannomas, artificial intelligence in the form of radiomics could play an important role because almost every patient will undergo magnetic resonance imaging (MRI).^3^ MRI images are traditionally interpreted subjectively by a clinician, with scrutiny on the tumour and its surrounding environment. However, modern advancements in technology have enabled quantitative and objective information such as size, texture and intensity to be extracted from MRI images. This can be seen as a reflection of the underlying tumour biology. The information obtained can then be analysed and associated with clinical outcomes. The process of image conversion into quantifiable data is known as radiomics.^4^ The use of radiomics involves four key steps: image acquisition, tumour segmentation, feature extraction and subsequent analysis (Figure 1).^5^

Radiomics has shown great promise in various specialties and diseases.^6^ In lung cancer, it has been accurate in predicting the malignancy of pulmonary nodules^7^ as well as both survival and response to chemotherapy and radiotherapy.^6^ In laryngeal cancer, radiomic analyses of baseline computed tomography (CT) imaging has been shown to predict survival outcomes.^5^

Despite this growing body of evidence in the use of radiomics, no study to date has critically reviewed the application of radiomics in vestibular schwannomas. Thus, this systematically informed review aims to evaluate the applications of radiomics in vestibular schwannomas, acknowledging the limitations and potential future uses.

## Method

### Search strategy and selection criteria

**Figure 2.**
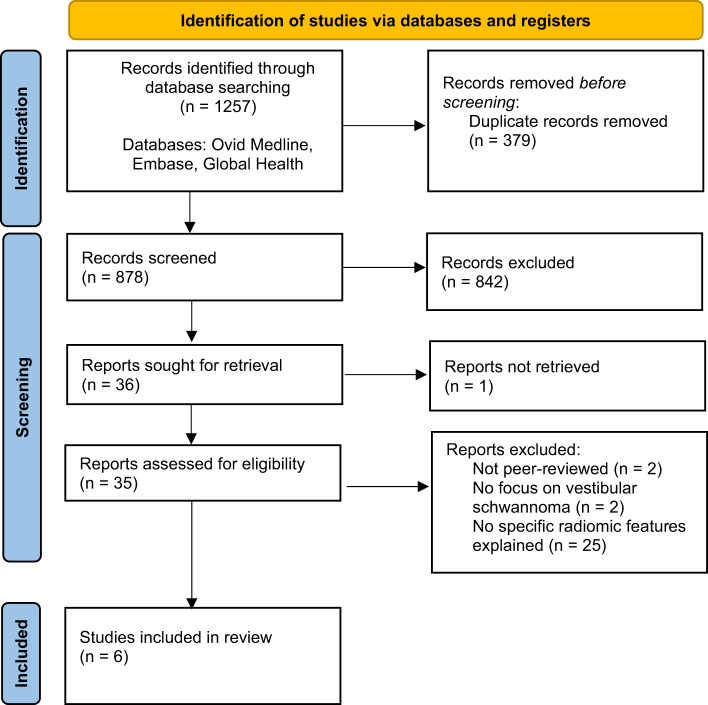
PRISMA Flowchart.

The review was pre-registered on Prospero (ID: CRD42024579319). The Preferred Reporting Items for Systematic Reviews and Meta-Analysis reporting guideline^8^ was used in manuscript preparation. A comprehensive systematic review-informed searchof the Ovid Medline, Embase and Global Health online databases was undertaken in January 2024. No time restriction was applied on the studies. Keywords ‘acoustic neuroma’ or ‘vestibular schwannoma’ or ‘cerebellopontine angle tumour’ or ‘cerebellopontine tumour’ or ‘head and neck cancer’ were combined with ‘radiomic’ or ‘signature’ or ‘machine learning’ or ‘artificial intelligence’. This resulted in 1257 studies. Duplicates were subsequently removed, abstracts were screened for relevance and only full-text, peer-reviewed articles in the English language were included. All peer-reviewed articles incorporating radiomic analysis of patients with vestibular schwannoma were included. This resulted in six studies (Figure 2)^8^ and the study findings are synthesised narratively.

## Results

Through the structured systematic search, 1257 studies were identified, of which 6 studies met the inclusion criteria. Findings from the included studies are detailed in Table 1 and in the following two sections.

**Table 1.** Summary of literature on the application of radiomics in AN

Author	Radiomics software used	Image modality	Study objective	Total number of AN patients (*n*)	Primary treatment	Study design	Model evaluation	Significant radiomic features	Conclusion
Bossi Zanetti *et al*.^11^	Pyradiomics (Python package)	MRI	To examine the prognostic properties of radiomic features extracted from pre-SRS contrast-enhanced T1-weighted MRI to predict vestibular schwannoma outcome at 24 and 36 months after SRS Cyberknife® treatment	108	SRS (linear accelerator)	Retrospective multicentre	External validation	At 24 months: HHL-median HHH-median LHL-minimum LLH-energy HHL-GLSZM-small area high grey-level emphasis HLH-GLSZM-high grey-level zone emphasis HHL-GLSZM-small area low grey-level emphasis HHH-GLSZM-zone entropy Original-GLCM-MCC HHL-GLCM-MCC At 36 months: LHL-skewness HHL-maximum LLH-energy HLH-kurtosis LLH−90 percentile HHH-GLSZM-zone entropy HHH-GLRLM-run entropy HHH-GLCM-cluster shade HHL-GLRLM-short-run high grey level emphasis HHL-GLSZM-small area high grey-level emphasis HHH-GLSZM-small area emphasis HHH-GLCM-MCC	Radiomic features can be used to predict tumour volume increase at 24 and 36 months after SRS
George-Jones *et al*.^14^	Pyradiomics version 3.0 + supervised linear SVM binary classification model	MRI	Determine if vestibular schwannoma shape and MRI texture features predict significant enlargement after SRS	53	SRS (gamma knife)	Retrospective single centre	Leave-one-out cross-validation	For larger tumours (>1.006 cm^3^): First-order texture uniformity First-order texture kurtosis GLCM maximum probability GLCM inverse normalised difference For smaller tumours (<1.006 cm^3^): Shape surface-volume ratio GLCM maximal correlational coefficient	A model involving radiomic features can predict enlargement of vestibular schwannoma post-SRS (AUC = 0.75)
Langenhuizen *et al*.^17^	GammaPlan version 11 + SVM via Matlab	MRI	Explore whether TTE post-GKRS can be predicted from the measured MRI tumour texture characteristics	99	SRS (gamma knife)	Retrospective single centre	10-fold cross-validation	GLCM entropy GLCM contrast GLCM energy GLCM correlation	TTE post-GKRS can be predicted from the measured MRI tumour texture features (sensitivity = 0.82, specificity = 0.69)
Narayanasamy *et al*.^16^	MimVista software version 6.6.5 (MIM Software Inc. Cleveland, OH, USA)	MRI	To determine whether radiomic features measured at baseline in MRI of vestibular schwannoma can predict GK treatment outcome	32	SRS (gamma knife)	Retrospective single centre	Nil	NGTDM complexity GLRLM run percentage	Two radiomic features extracted from baseline MRI of vestibular schwannoma can predict tumour volume post-SRS
Yang *et al*.^12^	Magnetic Resonance radiomics platform	MRI	To determine whether the radiomics analysis based on pre-GKRS MRI data could predict the transient tumour growth and long-term outcome of vestibular schwannoma post-GKRS	336	SRS (gamma knife)	Retrospective single centre	10-fold cross-validation	T1-weighted MRI: GLCM LLH cluster tendency GLCM cluster tendency Histogram HLL range T1 + contrast-weighted MRI: Texture HLL uniformity of LBP Histogram LLL minimum Histogram skewness Texture LLH mean of LBP GLRLM long-run low grey-level emphasis T2-weighted MRI: Histogram standard deviation GLRLM LLL long-run emphasis	Specific radiomic features could be used to train reliable machine-learning models for the forecast of tumour responses (i.e. transient growth) after GKRS
Song *et al*.^9^	Pyradiomics (Python package)	MRI	To explore the radiomics-based features MRI and construct a machine-learning model to predict the blood supply in vestibular schwannoma pre-operatively	191	Pre-surgery	Retrospective single centre	Five repeats, three-fold cross-validation	T1-weighted MRI: Contrast wavelet HHL first-order kurtosis Contrast square GLCM inverse variance Contrast square GLCM joint energy Contrast square root GLSZM size zone non-uniformity normalised Contrast wavelet HLL GLDM dependence variance Wavelet HLL GLRLM grey-level non-uniformity normalised T2-weighted MRI: Flair log sigma 3−0 mm, 3D first-order interquartile range Log sigma 3−0 mm, 3D GLRLM long-run high grey-level emphasis Exponential GLCM cluster prominence Flair log GLCM cluster shade Exponential GLDM dependence variance Exponential GLRLM short-run low grey-level emphasis	Machine-learning model outperforms the visual observation in ability to predict pre-operative blood supply (precision of model = 0.87, precision of doctor vision = 0.67)

AN = acoustic neuroma; MRI = magnetic resonance imaging; SRS = stereotactic radiosurgery; HLL/LLL/LLH refer to the types of wavelet filter applied to the MRI image axes, with H = high-pass filter and L = low-pass filter; GLSZM = grey-level size zone matrix; GLCM = grey-level co-occurrence matrix; MCC = maximal correlation coefficient; SVM = support vector machine; AUC = area under the receiver-operating characteristic curve; TTE = transient tumour enlargement; GKRS = gamma knife radiosurgery; GK = gamma knife; NGTDM = neighbourhood grey-tone difference matrices; GLRLM = grey-level run-length matrix; LBP = local binary pattern; GLDM = grey-level dependence matrix; 3D = three-dimensional.

### Radiomics for pre-operative decision-making

Only one study investigated radiomics for pre-operative decision-making in vestibular schwannomas. Song *et al*.^9^ aimed to explore the radiomics-based features of MRI and construct a model to predict the blood supply in vestibular schwannomas pre-operatively. The blood supply of vestibular schwannomas is an important factor affecting the complexity of surgery, and several studies have shown that an abundant blood supply is associated with facial nerve impairment and a decrease in clear resection rates.^10^ In the study, Song *et al*.^9^ conducted a retrospective review of 191 patients with histologically proven primary vestibular schwannoma and extracted several radiomic features. They then compared a radiomics-based model against subjective interpretation by experienced neurosurgeons in predicting tumour blood supply. The radiomics-based model had a precision of 0.87 against the neurosurgeons’ 0.67 in prediction of tumour blood supply. This study concluded that the employment of radiomics on baseline MRI images can be used as an effective method to predict the blood supply of a vestibular schwannoma. This approach also has better performance than a neurosurgeon’s judgement by visual observation of MRI images. Thus, it can provide information that can be essential when considering the operative approach. However, of note, this was a single-centre study and hence the results may not be generalisable.

### Radiomics for treatment outcomes

The remaining five studies in this review investigated the application of radiomics for treatment outcomes in vestibular schwannomas, with follow-up periods ranging from 6 months^11^ to 65 months.^12^ One of the ways in which a vestibular schwannoma is treated is with stereotactic radiosurgery, which employs beams to damage the DNA of targeted tumour cells.^13^ The affected cells then lose the ability to reproduce, which causes tumours to shrink.^13^

Bossi Zanetti *et al*.^11^ aimed to examine the prognostic properties of radiomic features extracted in pre-stereotactic radiosurgery MRI to predict vestibular schwannoma tumour volume at 24 and 36 months after stereotactic radiosurgery treatment. They conducted a retrospective, observational multicentre study (*n* = 108). They also performed a clinical-radiomic feature selection using the Least Absolute Shrinkage and Selection Operator, which is a statistical approach that prevents overfitting by highlighting the most significant features, and found 10 and 13 radiomic features at 24 and 36 months, respectively. These features were significant for predicting tumour volume at those respective time points. Hence, this suggests the potential of radiomic features to be used as predictors for treatment response.

George-Jones *et al*.^14^ sought to determine if the shape of a vestibular schwannoma and MRI radiomic (textural) features from pre-treatment MRI could predict significant enlargement after stereotactic radiosurgery. They defined the term ‘significant enlargement’ as being a 20 per cent increase in size as this is likely to have clinically relevant impact.^15^ They conducted a retrospective case review of 53 patients and found that the model they constructed from the tumour shape features and textural radiomic features demonstrated a sensitivity of 0.92 and a specificity of 0.65 in predicting whether a tumour would increase in size by more than 20 per cent after stereotactic radiosurgery. They also computed the area under the receiver-operating characteristic curve for three models. The area under the receiver-operating characteristic curve is a measure of the accuracy of a model. A value less than 0.5 is deemed as no better accuracy than chance, whilst a value with perfect accuracy is 1.^5^ The three models were a model for larger tumours, a model for smaller tumours and an overall model (i.e. including all tumours irrespective of size). The areas under the receiver-operating characteristic curves were 0.75, 0.65 and 0.76, respectively. Larger tumours referred to those with a volume greater than 1.006 cm^3^. Overall, this study shows radiomic analysis can effectively predict tumour enlargement, with good predictive performance. The study also suggests that the radiomics-based models were better at predicting enlargement in larger tumours compared with smaller tumours. The authors attributed this difference to the fact that a majority of the smaller tumours that they had used to train their model initially had already enlarged significantly post-stereotactic radiosurgery and hence there was limited training data available for this subgroup.^14^

Narayanasamy *et al*.^16^ sought to determine whether radiomic features from baseline MRI of vestibular schwannoma can predict gamma knife-based stereotactic radiosurgery treatment outcomes. They conducted a retrospective longitudinal study and investigated 32 patients, whereby they extracted 55 three-dimensional (3D) radiomic features from 3D pre-gamma knife MRI to quantify tumour characteristics. To measure treatment outcome, they defined a tumour volume increase of more than 10 per cent following gamma knife treatment to be a treatment failure. They then analysed each radiomic feature’s performance at successfully identifying a treatment failure through measurement of the area under the receiver-operating characteristic curve. They found that two radiomic features (neighbourhood grey-tone difference matrices and grey-level run-length matrices) displayed area under the receiver-operating characteristic curves of more than 0.65, suggesting potential in the capability of radiomic features to be used in the prediction of tumour outcomes post-gamma knife treatment.

Langenhuizen *et al*.,^17^ similarly to Narayanasamy *et al*.,^16^ aimed to explore whether transient tumour enlargement post-gamma knife treatment can be predicted from MRI tumour texture characteristics. They conducted a prospective single-centre study investigating 99 patients. They analysed scans obtained on the day of gamma knife treatment and at follow-up visits 6, 12, 24 and 36 months after treatment. They extracted MRI tumour radiomic texture features and found that first-order statistical MRI features (including mean, standard deviation, skewness, kurtosis and a 16-bin histogram) could not predict transient tumour enlargement. However, when utilising a particular set of radiomic features, namely four grey-level co-occurrence matrix features, the authors achieved a sensitivity of 0.82 and a specificity of 0.69, showing their prognostic value of transient tumour enlargement. For larger tumours (larger than 6 cm^3^), they obtained a sensitivity of 0.77 and a specificity of 0.89. This suggests that these four grey-level co-occurrence matrix features have the ability to predict transient tumour enlargement for smaller tumours with greater sensitivity but less specificity than for larger tumours. However, a notable limitation in this study is the retrospective nature of the study. The acquisition of scans on the day of gamma knife treatment is a strength because it prevents bias if there was growth between the initial scan and gamma knife treatment initiation.

Finally, Yang *et al*.^12^ aimed to explore whether the radiomics analysis of baseline MRI data could predict transient tumour growth and long-term vestibular schwannoma size post-treatment. They conducted a retrospective study using a longitudinal dataset of 336 patients, with each patient having a follow up at least 24 months post-radiosurgery. Three-dimensional low- and high-spatial frequency filters were applied on each MR modality to acquire multiscale representation, which aided in the subsequent extraction of the radiomic features. The authors extracted 1736 radiomic features from both T1- and T2-weighted MRI data pre-gamma knife treatment. T1 and T2 are different types of MRI scans, with T1 being useful in helping to highlight anatomy and T2 being helpful to identify pathology, such as inflammation.^18^ They found that the prediction of transient tumour growth achieved an area under the receiver-operating characteristic curve of 0.913 based on a set of five radiomic features. T1 features included contrast-enhanced low-pass, low-pass, high-pass filters mean of local binary pattern; high-pass, low-pass, low-pass filtersuniformity of local binary pattern and histogram low-pass, low-pass, low-pass filter sminimum. T2 features included grey-level run-length matrix low-pass, low-pass, low-pass filters long-run emphasis and histogram standard deviation. Using a set of another five radiomic features, the prediction of transient tumour growth achieved an area under the receiver-operating characteristic curve of 0.881. T1 features included grey-level co-occurrence matrix low-pass, low-pass, high-pass filters cluster tendency; contrast-enhanced grey-level run-length matrix long-run low grey-level emphasis and histogram skewness. T2 features included grey-level co-occurrence matrix cluster tendency and histogram high-pass, low-pass, low-pass filters range. Yang *et al*. concluded that the proposed machine-learning model that they had made based on the pre-gamma knife MRI radiomics provides the potential to predict transient tumour growth and long-term outcome of vestibular schwannoma post-gamma knife.^12^

## Discussion

This is the first critical review of the literature involving radiomics and vestibular schwannoma care. It demonstrates the potential for radiomic analysis from MRI to support pre-operative decision-making and predicting treatment response. All the studies that employed predictive models incorporating radiomic features had the potential to achieve accurate predictions. However, despite promising findings for the use of radiomics in vestibular schwannomas, there is a clear lack of studies investigating its application for this purpose. This is especially noted when investigating the application of radiomics for pre-operative decision-making, whereby the review only managed to identify one study. Despite the radiomics-based model in this study having a greater accuracy than the neurosurgeons,^9^ the single-centre nature of this study, alongside the fact that it is the only study that has investigated pre-operative tumour blood supply, resulted in a reduced generalisability of the findings.

All five studies that investigated radiomics in the prediction of outcomes of vestibular schwannomas suggested positive findings and the potential of radiomics to be used as predictors of outcomes post-radiosurgery. However, George-Jones *et al*.^14^ and Langenhuizen *et al*.^17^ showed different accuracies of radiomic analyses on smaller and larger tumours, with a smaller area under the receiver-operating characteristic curve and lower specificity for smaller tumours, respectively. George-Jones *et al*.^14^ explained the reduced area under the receiver-operating characteristic curve for smaller tumours, noting that the model used was trained with smaller tumours that had already significantly enlarged, leading to a reduced capability to detect smaller enlargements. Hence, this suggests the need for training data for the radiomics-based models to be composed of the most accurate and representative data possible to allow for accurate detection of relevant variables and prediction of outcomes such as tumour enlargement.

Furthermore, almost all the studies had a small number of patients investigated. Whilst this may be due to the rarity of the condition in the general population, a small sample size results in limited generalisability of findings and reduces the statistical power of the studies. This increases the risk of both type I and type II errors in the studies.

None of the radiomic features across the analyses were the same, suggesting that different radiomic features seem to be useful in different radiomics software and different patient cohorts. Only three studies employed the same radiomics software (Pyradiomics), but these studies noted different radiomic features as being significant. The use of radiomics software needs to be standardised and the Image Biomarker Standardisation Initiative will help this process.^19^

### Future considerations

This review has shown that radiomics has potential for application in vestibular schwannoma care. However, importantly, prospective multi-centre collaborative research and developments are required. Consensus on radiomics software used as well as scanning protocols also needs to be reached prior to considering translation into clinical practice. The Image Biomarker Standardisation Initiative is in a good position to do this, having published their first article in 2020 with the aim of providing standardisation for a set of 174 radiomic features.^20^

Furthermore, radiomics-based models should be externally validated. External validation is crucial for showcasing the strength and predictive capability of the model across independent datasets.^21^ These are critical determinants to clinical adoption.^21^

There are other avenues to consider for radiomic applications within vestibular schwannomas, such as facial nerve injury and/or complications. Furthermore, there should be a transition in care towards a ‘multi-omics’ approach because this is the future of personalised medicine. This involves taking an integrative approach that combines and analyses data sets of different ‘-omic’ groups such as genomics, epigenomics, transcriptomics, proteomics, metabolomics, radiomics and microbiomics^22^ of a patient. This will allow for correlation of all data available on a patient together and identification of patterns that could be missed in a single ‘-omics’ group. Integrating these approaches together, rather than individually, and correlating pathological clinical and imaging factors with a ‘multi-omics’ approach could be the key to making significant advancements in vestibular schwannoma management.

A key strength of this review was the comprehensive search strategy employed. In addition, the common pitfalls found across the various studies have been discussed. Thus, these limitations can serve as a roadmap for researchers developing new studies to ensure these issues are tackled. This review also focused on multiple aspects of vestibular schwannoma care and found literature supporting both the decision-making and post-operative stages of vestibular schwannoma care, although this was limited by the small amount of existing literature on this topic.

However, there are some limitations of the review. The variety in study designs resulted in a lack of statistical comparability, despite similar metrics being reported across multiple studies (i.e. sensitivity, specificity, area under the receiver-operating characteristic curve). Furthermore, the inclusion of only studies published in the English language could have resulted in some relevant studies not being included.

This review sets the platform for future research in this area. There needs to be larger, more collaborative studies with larger patient numbers. There is no value in conducting small single-centre studies. To achieve this goal, collaboration and uniformity regarding radiomic software and either the scanning parameters or robust harmonisation processes are needed. This will support future work in this area.

## Conclusion

This review highlights the great potential radiomics has in the treatment of vestibular schwannomas. Whilst there is a lack of literature around this topic, the evaluation of the existing literature provides a platform for further research, with the goal of eventually integrating radiomics in vestibular schwannoma care.

The lack of generalisable data, alongside the single-centre retrospective nature of majority of the studies, results in difficulty drawing accurate conclusions. However, the findings of the included studies are similar. Further large prospective studies, using more representative samples from multiple centres, alongside controlling for confounding variables, are required to further this field. To implement radiomics into clinical practice, a unified research effort is required.

## References

[1] Jackson C, and Creighton FX. *Acoustic Neuroma (Vestibular Schwannomas)*. Baltimore, Maryland, United States of America: Johns Hopkins Medicine Health 2023

[2] Mordor Intelligence. *Acoustic Neuroma Market Size & Share Analysis – Growth Trends & Forecasts (2024 – 2029)*

[3] Mayo Clinic. *Acoustic Neuroma: Diagnosis and Treatment*. Mayo Clinic United States: Mayo Clinic https://www.mayoclinic.org/diseases-conditions/acoustic-neuroma/diagnosis-treatment/drc-20356132; 2023

[4] Lambin P, Leijenaar RTH, Deist TM, Peerlings J, De Jong EEC, Van Timmeren J et al. Radiomics: the bridge between medical imaging and personalized medicine. *Nat Rev Clin Oncol* 2017;14:749–62 28975929 10.1038/nrclinonc.2017.141

[5] Rajgor AD, Patel S, McCulloch D, Obara B, Bacardit J, McQueen A, et al. The application of radiomics in laryngeal cancer. *Br J Radiol* 2021;94:20210499 34586899 10.1259/bjr.20210499 PMC8631034

[6] Arshad MA, Thornton A, Lu H, Tam H, Wallitt K, Rodgers N, et al. Discovery of pre-therapy 2-deoxy-2-18F-fluoro-D-glucose positron emission tomography-based radiomics classifiers of survival outcome in non-small-cell lung cancer patients. *Eur J Nucl Med Mol Imaging* 2019;46:455–66 30173391 10.1007/s00259-018-4139-4 PMC6333728

[7] Liu Y, Balagurunathan Y, Atwater T, Antic S, Li Q, Walker RC, et al. Radiological image traits predictive of cancer status in pulmonary nodules. *Clin Cancer Res* 2017;23:1442–9 27663588 10.1158/1078-0432.CCR-15-3102 PMC5527551

[8] Page MJ, McKenzie JE, Bossuyt PM, Boutron I, Hoffmann TC, Mulrow CD, et al. The PRISMA 2020 statement: an updated guideline for reporting systematic reviews. *BMJ* 2021;372:n71 33782057 10.1136/bmj.n71 PMC8005924

[9] Song D, Zhai Y, Tao X, Zhao C, Wang M, Wei X. Prediction of blood supply in vestibular schwannomas using radiomics machine learning classifiers. *Sci Rep* 2021;11:18872 34556732 10.1038/s41598-021-97865-5 PMC8460834

[10] Teranishi Y, Kohno M, Sora S, Sato H, Nagata O. Hypervascular vestibular schwannomas: clinical characteristics, angiographical classification, and surgical considerations. *Oper Neurosurg* 2018;15:251–61 29228328 10.1093/ons/opx246

[11] Bossi Zanetti I, De Martin E Pascuzzo R, D’Amico NC, Morlino S, Cane I, et al. Development of predictive models for the response of vestibular schwannoma treated with Cyberknife®: a feasibility study based on radiomics and machine learning. *J Pers Med* 2023;13:808 37240978 10.3390/jpm13050808 PMC10221826

[12] Yang H-C, Wu C-C, Lee C-C, Huang H-E, Lee W-K, Chung W-Y, et al. Prediction of pseudoprogression and long-term outcome of vestibular schwannoma after gamma knife radiosurgery based on preradiosurgical MR radiomics. *Radiother Oncol* 2021;155:123–30 33161011 10.1016/j.radonc.2020.10.041

[13] Hynes PR, Das JM. *Stereotactic radiosurgery (SRS) and stereotactic body radiotherapy* (SBRT). StatPearls Publishing, 2023 31194323

[14] George-Jones NA, Wang K, Wang J, Hunter JB. Prediction of vestibular schwannoma enlargement after radiosurgery using tumor shape and MRI texture features. *Otol Neurotol* 2021;42:e348–54 33065598 10.1097/MAO.0000000000002938

[15] Plotkin S, Halpin C, Blakely J, Slattery W, Welling DB, Chang S, et al. Suggested response criteria for phase II antitumor drug studies for neurofibromatosis type 2 related vestibular schwannoma. *J Neurooncol* 2009;93:61–77 19430883 10.1007/s11060-009-9867-7 PMC4036446

[16] Narayanasamy G, Zhang G, Siegel E, Campbell G, Moros EG, Galhardo EP, et al. Radiomic assessment of the progression of acoustic neuroma after gamma knife stereotactic radiosurgery. *J Solid Tumors* 2019;9:1

[17] Langenhuizen PPJH, Sebregts SHP, Zinger S, Leenstra S, Verheul JB, de With PHN. Prediction of transient tumor enlargement using MRI tumor texture after radiosurgery on vestibular schwannoma. *Med Phys* 2020;47:1692–701 31975523 10.1002/mp.14042 PMC7217023

[18] van Gastel, Maatje DA et al. T1 vs. T2 weighted magnetic resonance imaging to assess total kidney volume in patients with autosomal dominant polycystic kidney disease *Abdominal Radiology* 2018;43:1215–1222. doi:10.1007/s00261-017-1285-2 28871393 PMC5904223

[19] Zwanenburg, Alex et al. The Image Biomarker Standardization Initiative: Standardized Quantitative Radiomics for High-Throughput Image-based Phenotyping *Radiology* 2020;295. doi:10.1148/radiol.2020191145 PMC7193906 32154773

[20] Zwanenburg A, Vallières M, Abdalah MA, Aerts HJWL, Andrearczyk V, Apte A, et al. The image biomarker standardization initiative: standardized quantitative radiomics for high-throughput image-based phenotyping. *Radiology* 2020;295:328–38 32154773 10.1148/radiol.2020191145 PMC7193906

[21] Garau N, Paganelli C, Summers P, Choi W, Alam S, Lu W, et al. External validation of radiomics-based predictive models in low-dose CT screening for early lung cancer diagnosis. *Med Phys* 2020;47:4125–36 32488865 10.1002/mp.14308 PMC7708421

[22] Subramanian I, Verma S, Kumar S, Jere A, Anamika K. Multi-omics data integration, interpretation, and its application. *Bioinform Biol Insights* 2020;14:117793221989905 10.1177/1177932219899051 PMC7003173 32076369

